# The interplay between genetic background and sexual dimorphism of doxorubicin-induced cardiotoxicity

**DOI:** 10.1186/s40959-016-0013-3

**Published:** 2016-03-15

**Authors:** Beshay N. Zordoky, M. Judith Radin, Lois Heller, Anthony Tobias, Ilze Matise, Fred S. Apple, Sylvia A. McCune, Leslie C. Sharkey

**Affiliations:** 1 Department of Experimental and Clinical Pharmacology, University of Minnesota, 308 Harvard St S.E., Minneapolis, MN 55455 USA; 2 Department of Veterinary Biosciences, The Ohio State University, 1925 Coffey Road, Columbus, OH 43210 USA; 3 Department of Biomedical Sciences, University of Minnesota Medical School-Duluth, 1035 University Drive, Duluth, MN 55812 USA; 4 Veterinary Clinical Sciences Department, University of Minnesota, 1352 Boyd Ave, St. Paul, MN 55108 USA; 5 Veterinary Population Medicine Department, University of Minnesota, 1365 Gortner Ave, St. Paul, MN 55108 USA; 6 Department of Laboratory Medicine and Pathology, Hennepin County Medical Center and University of Minnesota, 701 Park Ave S, Minneapolis, MN 55404 USA; 7 Department of Integrative Physiology, University of Colorado at Boulder, 354 UCB, Clare Small 114, Boulder, CO 80309 USA

**Keywords:** Doxorubicin, Cardiotoxicity, Sexual dimorphism

## Abstract

**Background:**

Doxorubicin (DOX) is a very effective anticancer medication that is commonly used to treat hematological malignancies and solid tumors. Nevertheless, DOX is known to have cardiotoxic effects that may lead to cardiac dysfunction and failure. In experimental studies, female animals have been shown to be protected against DOX-induced cardiotoxicity; however, the evidence of this sexual dimorphism is inconclusive in clinical studies. Therefore, we sought to investigate whether genetic background could influence the sexual dimorphism of DOX-induced cardiotoxicity.

**Methods:**

Male and female Wistar Kyoto (WKY) and Spontaneous Hypertensive Heart Failure (SHHF) rats were used. DOX was administered in eight doses of 2 mg/kg/week and the rats were followed for an additional 12 weeks. Cardiac function was assessed by trans-thoracic echocardiography, systolic blood pressure was measured by the tail cuff method, and heart and kidney tissues were collected for histopathology.

**Results:**

Female sex protected against DOX-induced weight loss and increase in blood pressure in the WKY rats, whereas it protected against DOX-induced cardiac dysfunction and the elevation of cardiac troponin in SHHF rats. In both strains, female sex was protective against DOX-induced nephrotoxicity. There was a strong correlation between DOX-induced renal pathology and DOX-induced cardiac dysfunction.

**Conclusions:**

This study highlights the importance of studying the interaction between sex and genetic background to determine the risk of DOX-induced cardiotoxicity. In addition, our findings suggest that DOX-induced nephrotoxicity may play a role in DOX-induced cardiac dysfunction in rodent models.

## Background

The survival rate of cancer patients has substantially increased in the last decade due to advances in diagnosis and therapy [1]. It is estimated that approximately 14.5 million Americans with a history of cancer were alive in January 2014, and this number is expected to increase to nearly 19 million in 2024 [1]. Although increased survivorship is promising, these survivors may suffer from long term adverse effects of anticancer medications. Of importance, several anticancer agents may cause acute and/or delayed cardiotoxic side effects [2]. For instance, anthracyclines, such as doxorubicin (DOX), epirubicin, and daunorubicin, are very effective anticancer agents that are commonly used to treat both hematological malignancies and solid tumors [3]. Nevertheless, anthracyclines are known to have cardiotoxic effects that may progress to cardiac dysfunction and eventually heart failure [4]. Although the mechanism of DOX-induced cardiotoxicity is likely multi-factorial [5–10], the majority of studies support oxidative stress, inflammation, and cardiomyocyte apoptosis as being critical in the development of DOX-induced cardiomyopathy [11–13].

Several factors can increase the risk of cardiotoxicity after anthracycline treatment, including total cumulative dose, co-administration of other cardiotoxic agents, age, sex, and genetic predisposition to cardiovascular disease [14–17]. The effect of sex on DOX-induced cardiotoxicity in clinical studies is controversial. In some studies, male patients seem to be more susceptible to DOX-induced cardiotoxicity [18, 19]. On the contrary, other studies have shown that female sex is a risk factor for more severe DOX-induced cardiotoxicity [20]. In experimental studies, recent reports have demonstrated the protective effect of female sex against DOX-induced cardiotoxicity in Wistar and Spontaneously Hypertensive Rats (SHR) [21–24]. Since pre-existing cardiovascular disease is considered a significant risk factor for more severe DOX-induced cardiotoxicity [16] and we have shown that the genetic predisposition to cardiovascular diseases made male Spontaneous Hypertensive Heart Failure (SHHF) rats more susceptible to DOX-induced cardiac dysfunction than Wistar Kyoto (WKY) rats [25], we sought to investigate the interplay between genetic predisposition to cardiovascular diseases and sexual dimorphism in the context of DOX-induced cardiotoxicity.

The SHHF rat represents a genetic model of cardiomyopathy associated with hypertension, inflammation, and activation of the renin angiotensin aldosterone system which recapitulates the pathophysiology of heart failure in people [26]. Of interest, we have shown that sexual dimorphism occurs during the development of heart failure in SHHF rats with delayed onset of decompensated heart failure in female rats [27]. In the current study, we demonstrate for the first time that female sex is protective against DOX-induced cardiac dysfunction in SHHF rats. In addition, we show for the first time that female sex is protective against DOX-induced increase in systolic blood pressure in WKY rats. We also show that there is a strong correlation between DOX-induced renal injury and DOX-induced cardiac dysfunction, which highlights the importance of studying DOX-induced nephrotoxicity simultaneously with the well-known cardiotoxicity in rodent models.

## Methods

### Animals

All experimental procedures involving animals have been approved by the University of Minnesota Institutional Animal Care and Use Committee (IACUC). All animals were housed in an AAALAC accredited facility according to the NIH guidelines under controlled environmental conditions. All animals had free access to food and water during the study period. Thirteen male WKY rats (average weight 175 g), thirteen female WKY rats (average weight 135 g), fourteen male SHHF rats (average weight 160 g), and thirteen female SHHF rats (average weight 125 g) were obtained from Charles River Laboratories. All animals were 8 to 10-weeks old, and the SHHF rats were phenotypically lean. After a 1 week acclimation period, DOX rats received 8 weekly doses of 2 mg/kg DOX by subcutaneous injection (*n* = 7 for male WKY, female WKY, male SHHF, and female SHHF groups). Control rats received an equivalent volume of sterile saline solution (*n* = 6 for male WKY, female WKY, female SHHF; and *n* = 7 for male SHHF groups). The injection sites were periodically rotated to avoid repeated injections at the same site. Pharmacological grade DOX was purchased from Bedford Laboratories, Bedford, OH 44146. This protocol was determined based on preliminary studies to establish a model of delayed DOX-induced cardiotoxicity [25]. During the 8 weeks of the DOX administration phase and the 12 weeks following the last DOX injection, the delayed toxicity phase (a total of 20 weeks), rats were closely monitored for general health conditions as per the University of Minnesota IACUC guidelines. Animals were weighed at the beginning of the study (week 0), 4 weeks after the start of DOX (week 4), 8 weeks after the start of DOX (week 8), 6 weeks after the last DOX injection (week 14), and 12 weeks after the last DOX injection (week 20). Weight gain was calculated for each rat by subtracting its initial body weight from its weight at the following specified time points: 4, 8, 14, and 20 weeks after the first DOX injection. The animals were euthanized 12 weeks after the last DOX injections (week 20 of the study); the hearts and kidneys were carefully collected and weighed.

### Systolic blood pressure (SBP) measurement

SBP was measured by the tail cuff pressure method at the beginning of the study (week 0), 4 weeks after initiation of DOX (week 4), 8 weeks after the start of DOX (week 8), and 12 weeks after the last DOX injection (week 20). Briefly, rats were gently restrained in a warmed environment, and acclimated to the tail cuff pressure method. Thereafter, the SBP readings were taken using a BP-2000 Blood Pressure Analysis System™ for mice and rats (Visitech Systems, Inc., Apex, NC). The average of three stable SBP measurements was recorded. The change in SBP was calculated for each animal by subtracting the initial SBP from the SBP values at 4, 8, and 20 weeks after the first DOX injection.

### Echocardiography

The cardiac function was assessed by trans-thoracic echocardiography at 1, 6, and 12 weeks after the last DOX injection (weeks 9, 14 and 20 respectively). The echocardiography was performed by a board certified veterinary cardiologist (AT) who was blinded to strain, sex, and treatment group using an ATL 5000CV ultrasound system (Philips Medical Systems, Maplewood, MN). M-mode echocardiographic measurements included left ventricular internal diameter in diastole (LVIDd) and left ventricular internal diameter in systole (LVIDs). Fractional shortening (FS) was calculated as (LVIDd – LVIDs)/LVIDd × 100, as previously described [25].

### Serum cardiac troponin T measurement (cTnT)

Serum cTnT was measured 1 week after the last DOX injection using a commercially available third generation electrochemiluminescence immunoassay kit validated for rats, as per the manufacturer’s instructions (Roche Diagnostics, Indianapolis, IN).

### Histopathologic evaluation

Tissue sections were collected at the same level of the left ventricular free wall and the left kidney, fixed in 10 % neutral formalin, processed and embedded in paraffin using standard methods. Thereafter, four-micron tissue sections were cut and stained with hematoxylin and eosin (HE). Histopathologic evaluation was performed by a board certified veterinary pathologist (IM) in a blinded fashion to evaluate the following lesions in the heart: myocyte vacuolization and loss of myofibrils (according to the previously described scoring scheme [28]), myocyte necrosis characterized by coagulation necrosis and coagulative myocytolysis (Zenker’s necrosis), and interstitial proliferation and inflammation as described previously [25]. Briefly, each lesion was scored separately on 0–4 scale. Score 0 was given if no lesion was detected; 1 if the lesion was minimal; 2 if the lesion was mild; 3 if the lesion was moderate; 4 if the lesion was severe. The total score of myocardial damage was calculated as the sum of individual scores (range of possible score of 0–12). Similarly, the following lesions were assessed in each kidney: glomerular lesions, tubular lesions, and interstitial fibrosis and inflammation. Glomerular lesions as manifested by the presence of glomerulonephritis with increased mesangial matrix, mesangial or epithelial cell vacuolization, distention of capillary loops and adhesions between parietal and visceral podocytes; tubular lesions which included dilatation, protein casts, epithelial cell degeneration, atrophy and regeneration, and basement membrane thickening; and interstitial fibrosis and inflammation. Each lesion was scored separately on 0–4 scale and the total score of renal damage was calculated as the sum of individual scores (range of possible score of 0–12). as described previously [25].

### Statistical analysis

All statistical analyses were performed using the GraphPad Prism software (version 6.07, June 12, 2015). Results are presented as mean ± SEM. Comparisons among different sex and treatment groups was done by 2-way ANOVA, and comparisons involving multiple time points are done by 2-way ANOVA for repeated measures. Histopathologic grading of lesions is presented as median with 95 % confidence interval of the median. Statistical analysis for histopathologic grading was performed using Kruskal-Wallis non-parametric test. *P* values of <0.05 were considered statistically significant. Spearman rank correlations (r_s_) were used to examine associations between cumulative heart and kidney pathology scores and fractional shortening.

## Results

### Weight gain

**Fig. 1:**
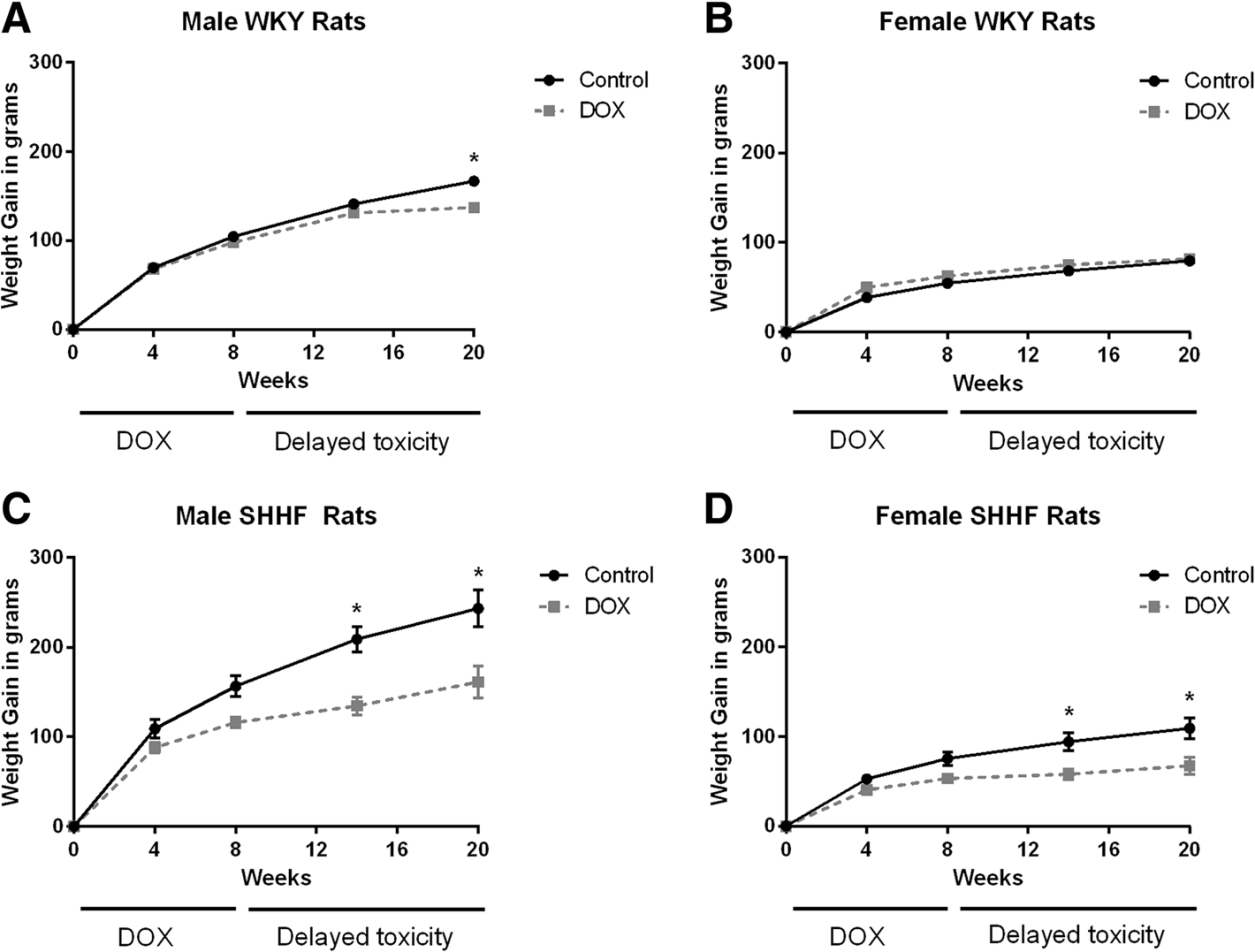
Body weight gain in male WKY rats **a**, female WKY rats **b**, male SHHF rats **c**, and female SHHF rats **d**. Weight gain was calculated for each rat by subtracting its initial body weight from its weight at the following specified time points: 4, 8, 14, and 20 weeks after the first DOX injection. * *p* < 0.05 significantly different from Saline group of the same sex

There was no significant morbidity or mortality in any of the experimental groups and all rats appeared healthy at the end of DOX administration and during the 12 weeks delayed toxicity phase. For male WKY rats, the weight gain was similar in control and DOX-treated animals at all time points except that DOX-treated rats gained less weight than the control at 20 weeks after the first DOX injection (12 weeks post-treatment) (Fig. 1a). For female WKY rats, DOX-treated animals gained the same weight during the DOX administration phase and during the 12 weeks delayed toxicity phase (Fig. 1b). On the other hand, both male and female DOX-treated SHHF rats gained less weight than their respective controls during the DOX administration and the delayed toxicity phases (Fig. 1c and d).

### Systolic Blood Pressure (SBP)

**Fig. 2:**
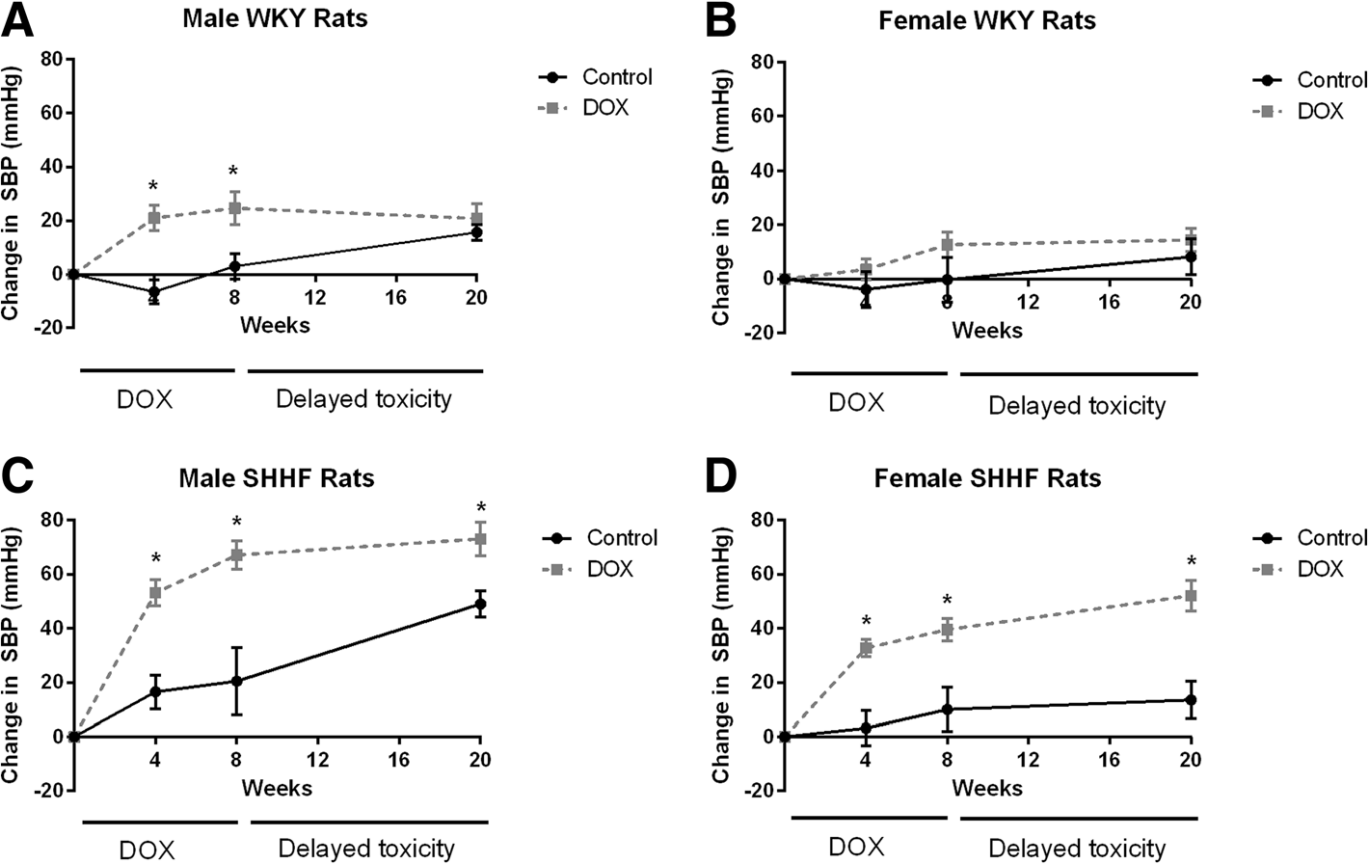
Change in Systolic Blood Pressure (SBP) in male WKY rats **a**, female WKY rats **b**, male SHHF rats **c**, and female SHHF rats **d**. SBP was measured by the tail cuff pressure method. The change in SBP was calculated for each animal by subtracting the initial SBP from the SBP values at 4, 8, and 20 weeks after the first DOX injection. * *p* < 0.05 significantly different from Saline group of the same sex

In male WKY rats, DOX treatment caused a significant increase in SBP during the DOX administration phase (Fig. 2a). Whereas, there was no significant difference in SBP between DOX-treated female WKY rats and their respective control (Fig. 2b). On the other hand, DOX treatment caused a significant increase in SBP in both male and female SHHF rats as compared to their respective control during both the DOX administration and the delayed toxicity phases (Fig. 2c and d).

### Serum cTnT levels

**Fig. 3:**
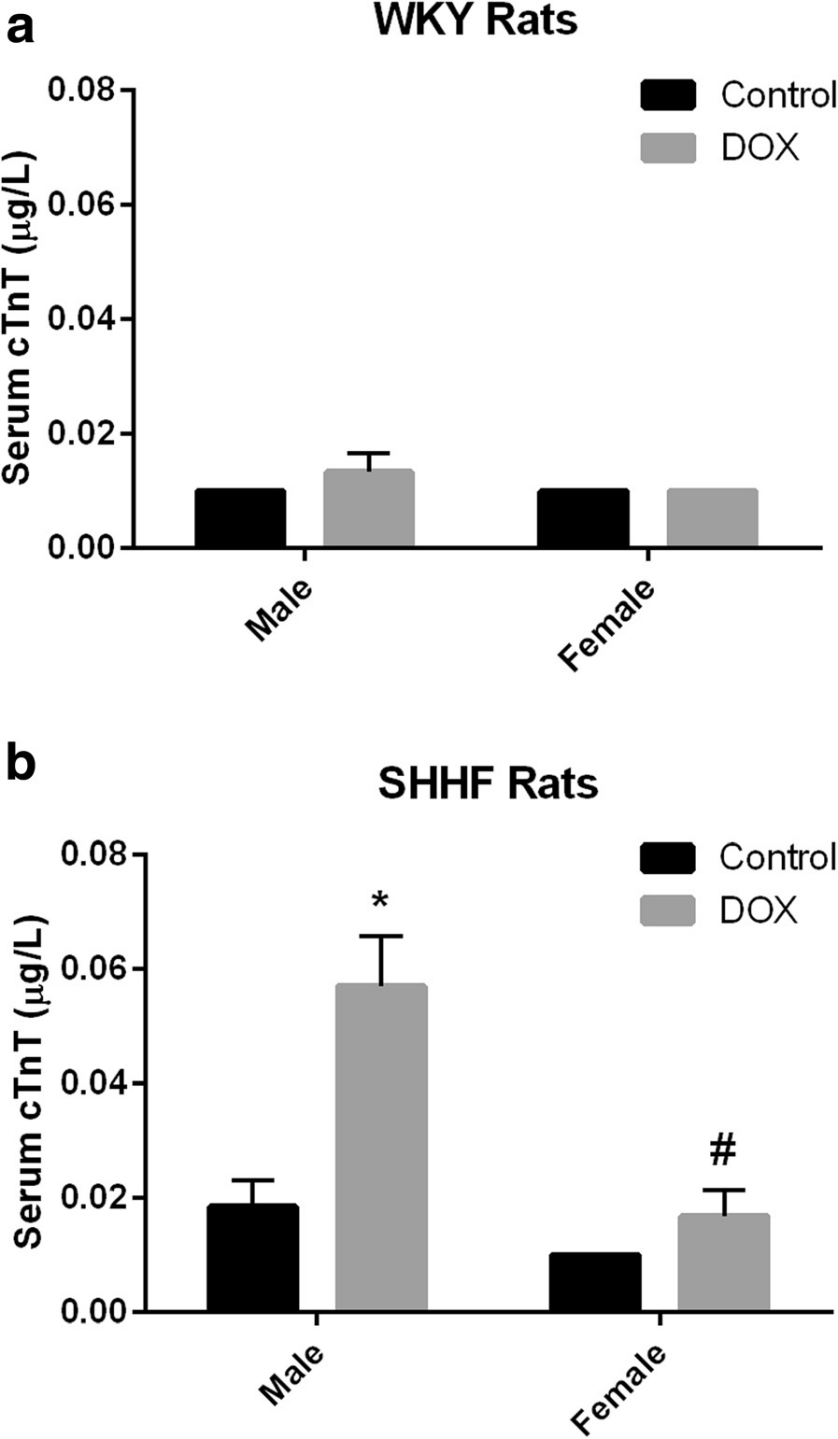
Serum cardiac troponin T (cTnT) in WKY rats (**a**) and SHHF rats (**b**). Serum cTnT was measured after 1 week of the last DOX injection. * *p* < 0.05 significantly different from Saline group of the same sex. # *p* < 0.05 significantly different from males within the same treatment group

There was no difference in the serum cTnT levels between the control and DOX-treated animals in both male and female WKY rats (Fig. 3a). On the other hand, serum cTnT level was about three-fold higher in DOX-treated male SHHF rats than their respective control. Whereas, there was no significant difference between DOX-treated and saline-treated female SHHF rats (Fig. 3b).

### Assessment of cardiac function

**Fig. 4:**
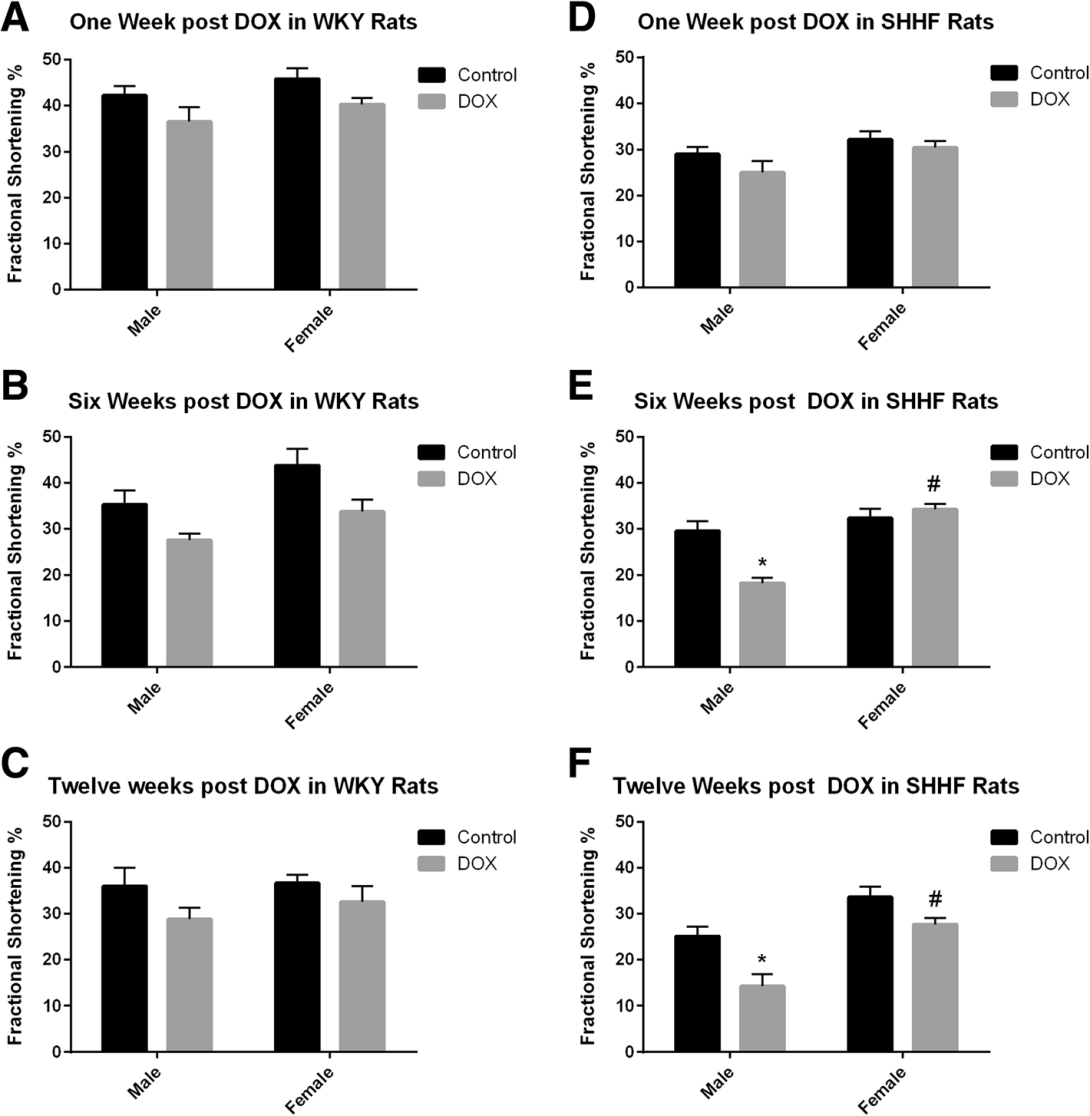
% Fractional Shortening (FS) 1, 6, and 12 weeks post-DOX in WKY rats (**a**, **b**, and **c**) and 1, 6, 12 weeks post-DOX in SHHF rats (**d**, **e**, and **f**). % FS was measured by trans-thoracic echocardiography.* *p* < 0.05 significantly different from Saline group of the same sex. # *p* < 0.05 significantly different from males within the same treatment group

The cardiac function was assessed in all the experimental groups by measuring the FS using trans-thoracic echocardiography 1,6, and12 weeks after the last DOX injection (weeks 5, 14 and 20 of the study, respectively). There was no significant difference in the FS between the control and DOX-treated animals at all time points in both male and female WKY rats (Fig. 4a, b, and c). Similarly, there was no significant difference in the FS between control and DOX-treated male and female SHHF rats 1 week after the last DOX injection (Fig. 4d). However, the FS was significantly lower in male DOX-treated SHHF rats than their respective controls at both 6 and 12 weeks after the last DOX injection. Interestingly, there was no significant difference in the FS between female DOX-treated SHHF and their respective controls at these time points (Fig. 4e and f).

### Heart weights and histopathology

**Fig. 5:**
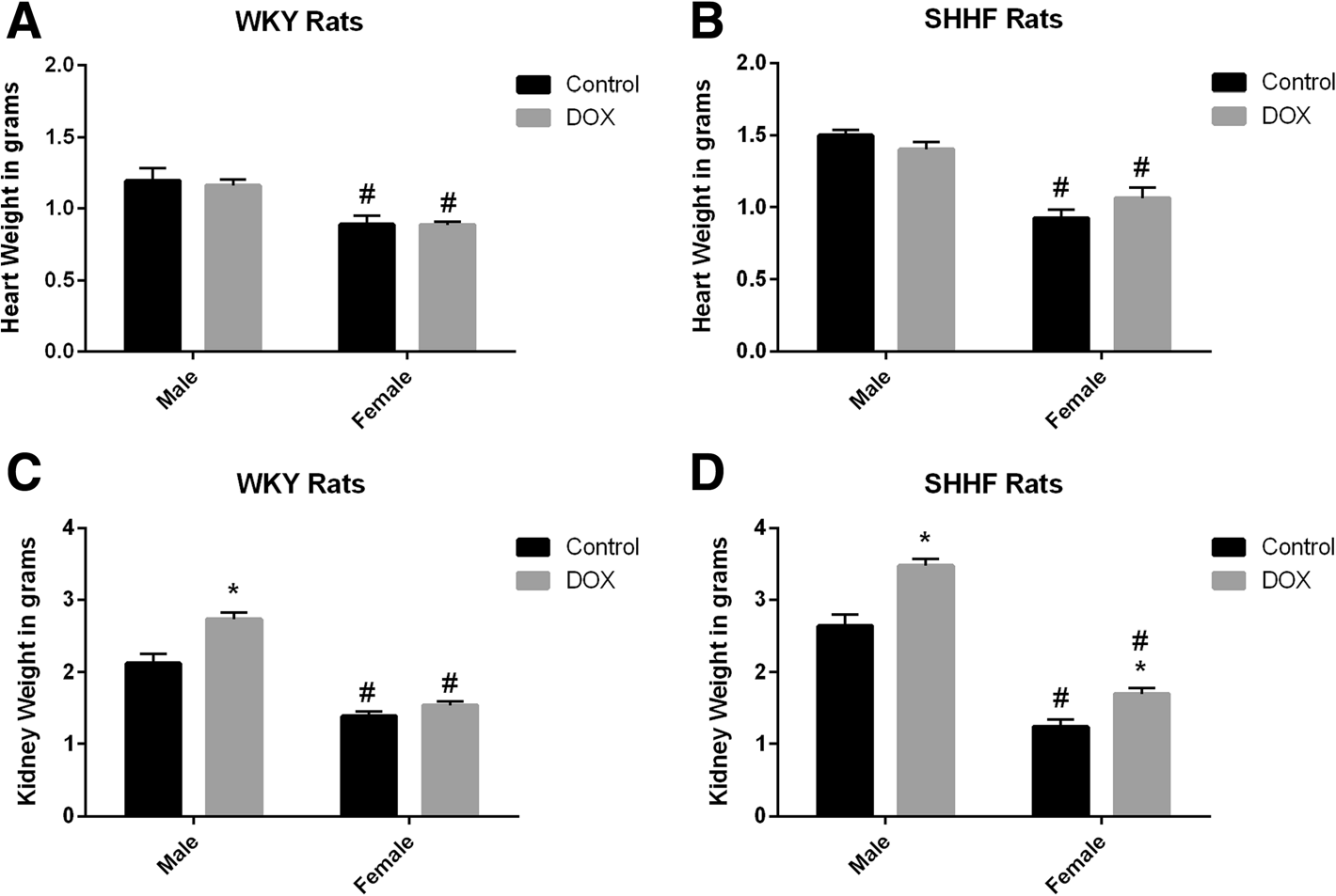
Total heart weight in WKY rats (**a**) and in SHHF rats (**b**), and total kidney weights in WKY rats (**c**) and in SHHF rats (**d**). The animals were euthanized 12 weeks after the last DOX injections (week 20 of the study); the hearts and kidneys were carefully collected and weighed. * *p* < 0.05 significantly different from Saline group of the same sex. # *p* < 0.05 significantly different from males within the same treatment group

There was no significant difference in the heart weight between DOX-treated animals and their respective controls in all experimental groups (Fig. 5a and b). DOX-treated male and female WKY rats had significantly higher myocyte vacuolization and cumulative heart pathology score than their respective controls. DOX-treated male WKY rats had significantly more severe interstitial cell proliferation and inflammation than their respective controls (Table 1). In SHHF rats, there was no significant difference in any cardiac lesion subtypes between DOX-treated male and female rats and their respective controls. Of interest, female rats had significantly lower interstitial cell proliferation and inflammation score than the male rats in the same treatment group (Table 2).

**Table 1:** Heart pathology scores in WKY rats based on evaluation of hematoxylin and eosin (HE) stained sections

Sex	Treatment	Myocyte vacuolization; loss of myofibrils score	Coagulation necrosis; coagulative myocytolysis score	Interstitial cell proliferation; inflammation score	Cumulative heart score
Male	Saline	1 (0–1)	0 (0–1)	0.5 (0–2)	2 (0–3)
	DOX	2 (2–3)*	1 (0–2)*	2 (1–3)*	5 (4–7)*
Female	Saline	0 (0–1)	0 (0–1)	0.5 (0–1)	1 (0–3)
	DOX	2 (2–3)*	1 (0–1)	2 (1–2)	5 (4–5)*

Data are presented as the median and 95 % confidence interval of the median

*p < 0.05 significantly different from Saline group of the same sex

**Table 2:** Heart pathology scores in SHHF rats based on evaluation of hematoxylin and eosin (HE) stained sections

Sex	Treatment	Myocyte vacuolization; loss of myofibrils score	Coagulation necrosis; coagulative myocytolysis score	Interstitial cell proliferation; inflammation score	Cumulative heart score
Male	Saline	1 (1–2)	1 (1–1)	2 (1–2)	4 (3–5)
	DOX	2 (1–2)	1 (0–2)	3 (1–3)	5 (3–7)
Female	Saline	1 (0–2)	0 (0–1)	0 (0–1)*	2 (0–3)
	DOX	1 (1–2)	1 (0–1)	1 (1–2)	3 (2–5)

Data are presented as the median and 95 % confidence interval of the median

*p < 0.05 significantly different from males within the same treatment group

### Kidney weights and histopathology

The kidney weights were significantly higher in DOX-treated animals than in their respective controls in male WKY rats, male SHHF rats, and female SHHF rats. There was no significant difference in the kidney weights between DOX-treated female WKY rats and their respective controls (Fig. 5c and d). All kidney lesions subtypes were significantly higher in male DOX-treated rats than in their respective controls. Female DOX-treated WKY had significantly lower interstitial inflammation and fibrosis than male DOX-treated rats of the same strain (Tables 3 and 4).

**Table 3:** Kidney pathology scores in WKY rats based on evaluation of hematoxylin and eosin (HE) stained sections

Sex	Treatment	Glomerular lesions score	Tubular lesions score	Interstitial inflammation and fibrosis score	Cumulative kidney score
Male	Saline	0 (0–0)	0 (0–0)	0 (0–0)	0 (0–0)
	DOX	2 (1–2)*	2 (1–3)*	2 (1–2)*	6 (3–7)*
Female	Saline	0 (0–0)	0 (0–0)	0 (0–0)	0 (0–0)
	DOX	1 (0–1)	1 (0–1)	0 (0–0)#	2 (0–2)

Data are presented as the median and 95 % confidence interval of the median

*p < 0.05 significantly different from Saline group of the same sex

#*p* < 0.05 significantly different from males within the same treatment group

**Table 4:** Kidney pathology scores in SHHF rats based on evaluation of hematoxylin and eosin (HE) stained sections

Sex	Treatment	Glomerular lesions score	Tubular lesions score	Interstitial inflammation and fibrosis score	Cumulative kidney score
Male	Saline	0 (0–0)	0 (0–0)	0 (0–0)	0 (0–0)
	DOX	4 (3–4)*	4 (3–4)*	3 (3–4)*	11 (9–12)*
Female	Saline	0 (0–0)	0 (0–0)	0 (0–0)	0 (0–0)
	DOX	2 (1–3)	2 (2–3)	2 (2–3)	6 (5–9)

Data are presented as the median and 95 % confidence interval of the median

*p < 0.05 significantly different from Saline group of the same sex

### Correlation between organ pathology and cardiac function

**Fig. 6:**
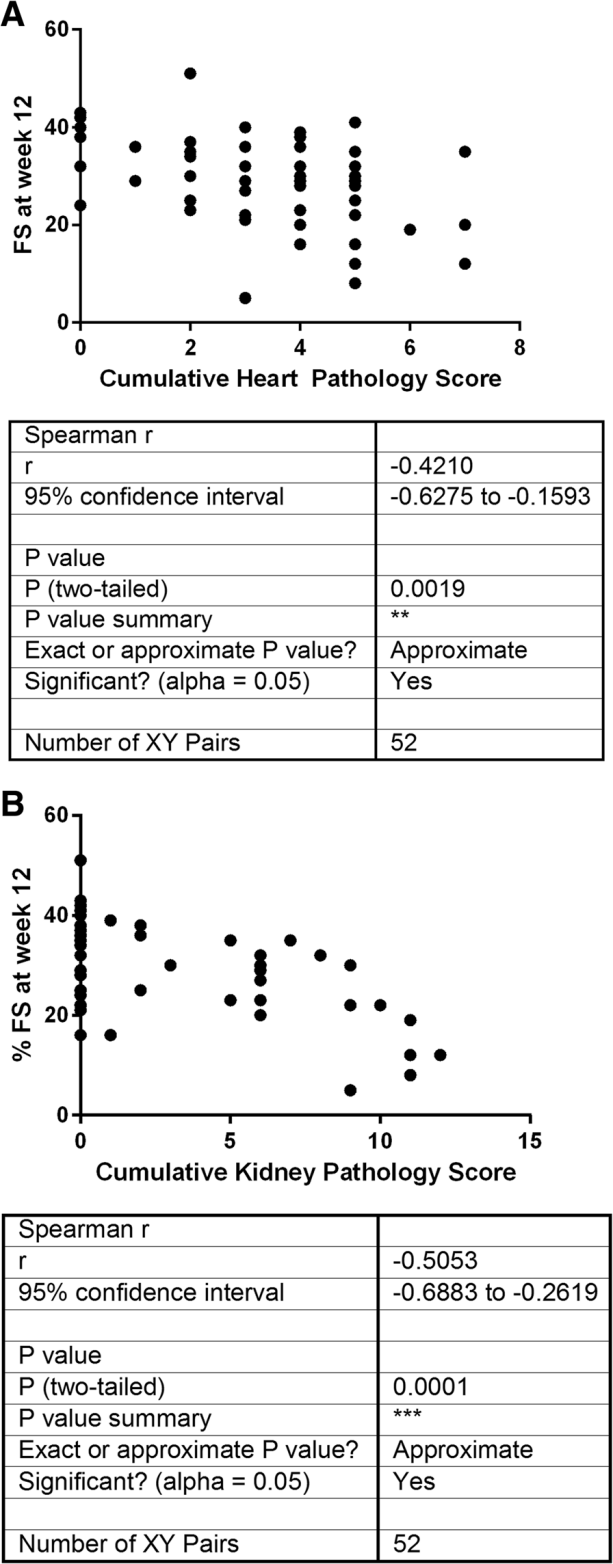
Correlation between cumulative heart pathology score (**a**) or kidney pathology scores (**b**) and % fractional shortening (FS). The total cumulative heart and kidney pathology scores based on evaluation of hematoxylin and eosin (HE) stained sections were correlated with the % FS measured by trans-thoracic echocardiography for each rat. Nonparametric Spearman correlation was used to assess the degree and significance of the correlation

In order to assess the relationship between DOX-induced organ damage and DOX-induced cardiac dysfunction, we evaluated the correlation between both the heart and the kidney pathology scores and the FS. There was a significant inverse correlation between the cumulative heart pathology score and the fractional shortening with an r of only -0.4210 (p value of 0.0019) (Fig. 6a). Surprisingly, the correlation between the cumulative kidney pathology score and the fractional shortening was stronger with an r of -0.5053 and a p value of 0.0001 (Fig. 6b).

## Discussion

It has been widely accepted that the risk of cardiovascular diseases is lower in women than in men [29]. Nevertheless, women may suffer poorer outcome after the occurrence of an adverse cardiovascular event [30]. Although there is a plethora of studies that address the issue of sexual dimorphism in several cardiovascular diseases, there is inconclusive and scarce information about sexual dimorphism in chemotherapy-induced cardiotoxicity [31]. For instance, female sex has been considered a risk factor for delayed DOX-induced cardiotoxicity in pediatric cancer patients [15]. Similarly, female breast cancer patients are very susceptible to the cardiotoxic adverse effects of DOX, especially when combined with other cardiotoxic agents such as trastuzumab or radiation [32, 33]. On the other hand, some studies in middle age patients have demonstrated that male patients are more susceptible to DOX-induced cardiotoxicity than female patients [18, 19]. Indeed, the aforementioned findings suggest that other factors such as age, menopausal state, and genetic predisposition to cardiovascular diseases may alter the protective effect of female sex against DOX-induced cardiotoxicity. Therefore, the purpose of this study is to examine the effect of genetic predisposition to cardiovascular diseases on sexual dimorphism of DOX-induced cardiotoxicity.

In the present study, we have adopted a clinically relevant regimen of 8 weekly doses of 2 mg/kg DOX [25]. This dosage regimen has been thoroughly optimized to allow a prolonged follow-up of the experimental animals up to 12 weeks after the last DOX injection without any mortality. This long follow-up period has allowed us to document sex-related differences of early and delayed DOX-induced toxicity in both the normotensive WKY rats, and the hypertensive heart failure prone SHHF rats. DOX administration began when the average age of the rats was 10 weeks. At this age, there is no overt cardiomyopathy in SHHF rats [27]. Therefore, the aim of this study is to model cancer patients with no current cardiovascular diseases, but with a genetic background that may exacerbate a cardiovascular disease as part of a delayed response to DOX. Since weight loss is one of the hallmarks of DOX-induced toxicity in experimental animals [10], we assessed the weight gain in all saline and DOX-treated rats during both the DOX administration phase and the follow-up phase. In the current study, female sex was protective against DOX-induced weight loss in WKY rats. In another study, DOX treatment caused a significant weight loss in both male and female Wistar rats [22]. Indeed, the degree of DOX-induced toxicity was much higher in that study than in our current study. For instance, there was a significant 50 % mortality in male DOX-treated rats in that particular study [22], whereas there was no mortality in ours. In SHHF rats, both male and female DOX-treated rats gained less weight compared to their respective controls. Similar to our findings, there was a significant weight loss in both male and female DOX-treated SHR rats as compared to their respective controls [23]. These findings may indicate that genetic predisposition to cardiovascular diseases in both SHR and SHHF has negated the protective effect of female sex against DOX-induced weight loss.

Although hypertension is more prevalent among cancer survivors than the general population [34], the effect of DOX-induced toxicity on blood pressure is inconclusive. DOX-induced toxicity has been reported to lower the blood pressures in some studies [35, 36], and to increase the blood pressure in other studies [37, 38]. In the current study, we demonstrate that DOX causes a significant increase in SBP during the DOX administration period in male WKY rats. However, there was no significant difference in SBP between DOX-treated male WKY rats and their respective control 12 weeks after the last DOX injection. There was no difference in SBP between DOX-treated female WKY rats and their respective controls both during the DOX administration period and during the delayed toxicity phase. DOX-induced increase in blood pressure can be attributed to the known DOX-induced nephrotoxicity and vascular toxicity; both are known regulators of blood pressure [39, 40]. To the best of our knowledge, this is the first report that female sex is protective against DOX-induced increase in SBP in the normotensive WKY rats. However, female sex has been shown to protect against hypertension in other models. For instance, it has been shown that female sex protects against angiotensin II-induced elevation of blood pressure in human subjects [41]. On the other hand, DOX-induced toxicity caused a significant increase in SBP in both male and female SHHF rats than their respective controls during the DOX administration and the follow-up periods. These findings demonstrate that the protective effect of female sex against DOX-induced elevation of blood pressure may be modulated by the genetic pre-disposition of the treated subjects.

To investigate the effect of sex and strain differences on DOX-induced cardiotoxicity, we measured serum cTnT which has been widely used as a biomarker for DOX-induced cardiotoxicity [25, 42]. The increase in cTnT usually precedes the deterioration of ventricular function [43]. In the current study, there was no significant change of the serum cTnT level in both male and female WKY rats when measured 1 week after the last DOX injection. Similarly, DOX administration was not associated with a significant decline in the cardiac function in either male or female WKY rats. On the other hand, DOX administration caused a significant increase in the cTnT level in male SHHF rats, whereas there was no difference in female SHHF rats. Similarly, there was a significant decline in the cardiac function in male SHHF rats when measured during the delayed toxicity phase, but not in female SHHF rats. These findings demonstrate for the first time the protective effect of female sex against DOX-induced cardiotoxicity in SHHF rats. In addition, these results confirm that the genetic pre-disposition to cardiovascular disease in male SHHF rats can aggravate DOX-induced cardiotoxicity.

Several mechanisms have been proposed to explain the protective effect of female sex against DOX-induced cardiotoxicity. Mitochondrial dysfunction, energy metabolism, and cardiolipin remodeling have been proposed as critical aspects of sex differences of DOX-induced cardiotoxicity in Wistar rats [22, 24]. In SHR, sex-specific differences in mast cell activity and mitochondria-related oxidative stress gene expression have been suggested as potential mechanisms for sex differences in DOX-induced cardiotoxicity [21, 23]. It has also been proposed that estrogen plays the main protective role against DOX-induced cardiotoxicity in female animals, since ovariectomized female animals become as sensitive to DOX-induced cardiotoxicity as male animals [21, 23]. Similarly, ovariectomy has been shown to exacerbate DOX-induced cardiotoxicity in female Wistar rats due to diminished antioxidant capacity in the ovariectomized animals [44]. Other mechanisms of the cardioprotective effects of estrogen include anti-apoptotic [45], anti-fibrotic [46], and anti-hypertrophic effects [47]. Of interest, we have demonstrated that sexual dimorphism plays a role in the progression into decompensated heart failure in SHHF rats with male rats developing earlier activation of the renin angiotensin aldosterone system and earlier decline in their cardiac function [27]. The exact mechanisms by which female sex protects against DOX-induced cardiotoxicity in SHHF rats have yet to be fully explored.

In addition to the well-recognized DOX-induced cardiotoxicity, DOX causes significant nephrotoxicity characterized by chronic proteinuria, glomerular sclerosis, and interstitial and tubular involvement primarily in rodent models [48–50]. Similar to DOX-induced cardiotoxicity, oxidative stress is thought to play the major role in mediating DOX-induced nephrotoxicity [51]. Interestingly, DOX-induced nephrotoxicity has also been reported to be less severe in females than in males of SHR and Sprague–Dawley rats [21, 52]. Male sex hormones have been shown to worsen DOX-induced nephrotoxicity, whereas female sex hormones play a protective role [52]. Since there was a strain difference in DOX-induced nephrotoxicity between male WKY and SHHF rats [25], we sought to investigate the sexual dimorphism of DOX-induced nephrotoxicity in both WKY and SHHF rats. In agreement with previous studies, DOX-induced kidney damage was less severe in female WKY rats than in males of the same strain. Interestingly, we demonstrate for the first time that DOX-induced kidney damage was also less severe in female SHHF rats than in males of the same strain. DOX-induced kidney damage was more severe in male and female SHHF rats than in WKY rats of the same sex. These findings demonstrate that the extent of DOX-induced nephrotoxicity is determined by the interplay between genetic background and sexual dimorphism.

To our surprise, the extent of sexual dimorphism was more prominent in DOX-induced pathological lesions in the kidney than those in the heart. Indeed, DOX-induced cardiotoxicity did not cause any significant increase in the heart pathology scores in either male or female SHHF, despite the fact that DOX-induced cardiotoxicity caused a significant decline in cardiac function in male SHHF. This discrepancy between DOX-induced cardiac pathology and DOX-induced cardiac dysfunction suggests the possibility that other non-cardiac pathology may play a part in mediating and/or worsening of DOX-induced cardiac dysfunction primarily in rodent models. Since the cardiovascular-renal axis is an important factor in cardiovascular health and disease [53], we sought to investigate the relationship between kidney pathology and cardiac function in our study. We discovered that kidney pathology scores are strongly correlated with the decline in cardiac function. Thus, DOX-induced nephrotoxicity may have exacerbated DOX-induced cardiac dysfunction in the current study. Indeed, DOX-induced nephrotoxicity is a factor that may be overlooked in rodent studies of DOX cardiotoxicity, so characterization of the renal damage as a component of the effects of DOX is important physiologic context for interpretation of the heart data from these studies. In addition, these findings may have clinical implications in renally-impaired cancer patients who may become more susceptible to the cardiotoxic effects of DOX. In support of this concept, renal dysfunction has been shown to increase the risk of chemotherapy-induced cardiotoxicity in cancer patients [54, 55]. A limitation of this study is that DOX-induced nephrotoxicity was assessed only by kidney histopathology. Other tests including creatinine clearance and proteinurea are required to better assess the renal function. In addition, DOX-induced nephrotoxicity may have contributed to the observed increase in cTnT in DOX-treated male SHHF rats due to decreased clearance of cTnT fragments [56]. Therefore, further research is required to identify the mechanisms by which nephrotoxicity can alter the pathogenesis of DOX-induced cardiotoxicity in rodent animal models.

## Conclusions

In the current study, we demonstrated that genetic background influences the sexual dimorphism of DOX-induced toxicity. Female sex is protective against DOX-induced weight loss and DOX-induced increase in blood pressure in the normotensive WKY rats, and it is protective against DOX-induced cardiac dysfunction in the hypertensive heart failure prone SHHF rats. In both strains, female sex was protective against DOX-induced nephrotoxicity. Therefore, this study highlights the importance of studying the interaction between sex and genetic background to determine the risk of DOX-induced cardiotoxicity. In addition, our findings suggest that DOX-induced nephrotoxicity may play a role in worsening DOX-induced cardiac dysfunction in rodent models.

## Abbreviations

cTnT: cardiac troponin T
DOX: doxorubicin
FS: fractional shortening
HE: hematoxylin and eosin
SBP: systolic blood pressure
SHHF: spontaneous hypertensive heart failure
SHR: spontaneously hypertensive rat
